# Pivoting to stay the course: How women entrepreneurs take advantage of opportunities created by the COVID-19 pandemic

**DOI:** 10.1177/0266242620949136

**Published:** 2020-09

**Authors:** Tatiana S Manolova, Candida G Brush, Linda F Edelman, Amanda Elam

**Affiliations:** Bentley University, USA; Babson College, USA; Bentley University, USA; Babson College, USA

**Keywords:** business models, COVID-19, crisis, women entrepreneurs

## Abstract

COVID-19 is unique in the severity of its impact as it is a humanitarian disaster that has caused both a supply and a demand shock to the global economic system. It has disproportionately affected women entrepreneurs as their firms are younger and smaller. In this commentary, we contend that while all businesses must pivot their business models in times of tumultuous change, simultaneously reducing risk and seizing new opportunities, this is particularly difficult for women entrepreneurs, whose businesses are concentrated in the industry sectors most severely affected by the economic shutdown. We draw on recent survey data from the Diana International Research Institute (DIRI) to identify business model pivots in women-owned businesses, and conclude by offering a set of gendered future research questions.

‘As with many things, the pandemic will reveal many things about the American economy and shape its future. This COVID-induced “she-cession” has important implications’

(Hadley Heath Manning, The Hill, 2020)

In this commentary, we systematise our observations on how women entrepreneurs are pivoting their business models in response to the COVID-19 pandemic. COVID-19 is unique in the severity of its impact as it is a humanitarian disaster that has caused both a supply and demand shock to the global economic system. As such, it provides the perfect, albeit costly, natural experiment to examine successful business model pivots in response to a crisis that is both economic and humanitarian. We challenge a dominant narrative in entrepreneurship research, which categorises crisis response into a stereotypical gendered dichotomy: women seeking to reduce risks and men seeking to seize opportunities (Jianakoplos and Bernasek, 1998; Maxfield et al., 2010). In contrast, we argue that (1) to understand business models, we must take into consideration the economic and social structure in which businesses operate and (2) that successful business model pivots, particularly in response to double shocks such as those inflicted by COVID-19, must *simultaneously* reduce risk and seize opportunities (McGrath and Macmillan, 2009). We support our contentions with evidence from two case studies and data from the Diana International Research Institute’s (DIRI) series of surveys of women entrepreneur’s response to the COVID-19 pandemic and offer several directions for further research into business model pivoting in response to complex exogenous shocks.

## Business models

In its most basic form, the business model provides an outline of the ways in which a firm creates and delivers value to its customers (Seddon et al., 2004). While researchers offer different definitions (see Wirtz et al., 2016, for a current review), there is a strong consensus in the literature that the business model encompasses customer-focused value creation, a profit formula, key resources and key processes (Casadesus-Masanell and Ricart, 2011; Teece, 2010). This consensus suggests business models can be a source of innovation and competitive advantage (Christensen, 2001; Zott and Amit, 2008). But, once a business model is determined, it is subject to change. A number of forces trigger business model adjustments, including technology, external stakeholders, performance, regulatory forces, market forces or exogenous shocks (Saebi et al., 2017). The effects of business model changes most commonly involve innovation in resources, offering, customers and finances, while the process involves adaptation or pivoting (Osterwalder and Pigneur, 2010).

McGrath (2010) proposed that in highly uncertain, complex and fast-moving environments, businesses should adopt a discovery-driven approach to their business model and move from analysis and execution to continuous and controlled experimentation and learning. In discovery-driven business models, the focus is on the market opportunities and on the nimble deployment of resources that lead a firm to enjoy superior efficiency or effectiveness on the key variables that influence its profitability (McGrath, 2010). In other words, a discovery-driven approach to business model pivoting implies reducing risks, while simultaneously seizing opportunities. This dual focus challenges traditional gendered conceptions of crisis response in which women seek to reduce risk, while men seek to seize opportunities, as we discuss next.

## Business models and gendered crisis response: what does the literature tell us?

To date, very few studies focus specifically on gender differences in business recovery or business model pivoting following natural disasters or economic shocks (Li et al., 2019; Marshall et al., 2015; Young et al., 2017). Some literature suggests that women and men respond differently to stress and external shocks by managing their firms differently (Bradshaw, 2013; Young et al., 2017), while others suggest that gender differences in survival are better explained by the different types of businesses that men and women own and manage (Fothergill, 1996; Kalnins and Williams, 2014; Marshall et al., 2015). In a study of firms recovering from Hurricane Andrew, Morrow and Enarson (1996) found that women-owned businesses experienced effects that were more adverse during the recovery period. After the Wenchuan earthquake in New Beichuan, China, Li et al. (2019) found that male business owners were more likely to succeed in continuously operating their businesses than female owners. Following Hurricane Katrina, Marshall et al. (2015) found that businesses owned by women were more likely to fail than those owned by men, and that larger and older businesses, service-based businesses, and those run by owners with more industry experience and prior disaster experience, were less likely to fail.

Research findings from studies investigating gender differences in preparedness, responsiveness and benefits from business relief are also mixed. In a study of the application process for federal disaster loans following Hurricane Katrina, Josephson and Marshall (2016) found that women owners were more likely to apply for disaster loans but, on average, received lower amounts. Hiramatsu and Marshall (2018) found that receiving a small business administration (SBA) disaster loan after Hurricane Katrina played a positive and statistically significant role in determining the actual revenue change and owner perception of revenues, but only for male owners.

Following the 2008 financial crisis, one study concluded that women entrepreneurs dealt with the resulting recession in a defensive manner, while men used an offensive approach (Cesaroni et al., 2015). Another study of the global financial crisis found that women had lower demand for bank loans but were more successful, concluding that feminised risk aversion might inform the more conservative approach (Cowling et al., 2019). Alternatively, a comparative study of sole proprietors during the financial crisis showed women personally take care of house and family, and therefore, had more difficulty with work–family conflict and internal psychological balance than their male peers (Cesaroni et al., 2018). In sum, prior research has suggested that women tend to be more adversely affected by economic downturns and natural disasters and are more likely to adopt a defensive crisis response stance.

## COVID-19’s impact on business models

As we consider COVID-19’s impact on entrepreneurial business models, we see an extreme exogenous shock and its immediate impact upon existing businesses. It is important to note that not all ‘external shocks’ are equal in force, as they do not ‘elicit or deserve the same response with the same effects from given agents’ (Davidsson, 2019: 2). In fact, some external shocks are gradual and/or reasonably predictable, while others are traumatic and unpredictable (Davidsson, 2019; Davidsson et al., 2018). In addition to being unpredictable and dramatic, and unlike prior exogenous shocks, the COVID-19 pandemic caused a double shock to the global economic system. On the supply side, COVID-19 resulted in the immediate cessation of contact-intensive business sectors affected by the lockdown policies and in large-scale disruptions in global supply chains. On the demand side, COVID-19 led to abrupt rises in demand for certain classes of hospital equipment and supplies, personal protection equipment, household staples and digital services, while simultaneously causing a dramatic drop in demand for services such as non-emergency healthcare, bars, restaurants, entertainment or travel and accommodation services. Because of this unprecedented pandemic, assumptions forming the basis of current business models were rapidly overturned, as all aspects of current operating certainty disappeared. This change in circumstances paved the way for new assumptions and business projections and called for a thorough rethinking and radical pivoting of established business models.

## COVID-19’s impact on women entrepreneurs

While early clinical evidence shows men are more heavily affected by the health effects of the COVID-19 pandemic (Curley, 2020), the resulting economic crisis is disproportionately affecting women entrepreneurs (UN Women, 2020; WE Forum, 2020; Werner, 2020). Key structural differences need to be considered when assessing the types of crisis response and business model pivoting women entrepreneurs undertake. In fact, we argue that business model pivots cannot be fully understood without taking into consideration the economic and social structure in which businesses operate.

Not only are women-owned businesses typically more vulnerable to economic impacts due to lower average firm age and size, but also they are concentrated in the industry sectors hit hardest by economic shutdowns (Kalnins and Williams, 2014; McManus, 2017; WE Forum, 2020). Recent data from the Global Entrepreneurship Monitor show that more than 50% of women entrepreneurs operate in the wholesale/retail trade sector, compared to 42.6% of men, and 17.2% of women operate in government/health/education and social services compared to 10.1% of men (Elam et al., 2019). These sectors are characterised by lower entry barriers, have a heavy reliance on consumers as customers (rather than businesses) and are extremely competitive, which makes them among the most vulnerable in most economies. Furthermore, in the absence of school, childcare or eldercare services, women entrepreneurs bear the majority of the burden of family care (OECD, 2020). In addition, women also take on a greater proportion of housework (Jackson, 2019). This creates a perfect storm for women entrepreneurs. While business relief programmes offer some buffer against environmental shocks, policy responses typically are directed to all entrepreneurs and do not take gender into consideration, which leaves women entrepreneurs to fend for themselves in many respects (Bradshaw, 2013; Brush and Greene, forthcoming; Cupples, 2007).

In sum, the COVID-19 pandemic has caused the following three major challenges for women entrepreneurs: (1) the industries where most women operate are disproportionately affected by the recession; (2) women are more likely to run many of the youngest, smallest, most vulnerable businesses; and (3) with schools closed and elderly family members under threat, women are more likely to be juggling primary care-giving and homemaking, while they are scrambling to save their businesses. The prospects are daunting, and the options for women are limited. However, the solutions to these gender-linked challenges for women entrepreneurs may include both cost-cutting and pivoting to capture new business opportunities presented by the crisis.

## Challenges related to COVID-19 for women entrepreneurs: findings from Diana International Research Institute surveys

The DIRI at Babson College conducted a series of four surveys beginning in April 2020, to explore the impact of the COVID-19 pandemic crisis and subsequent lockdown on women business operations and to get their perspective. Results from the first survey suggest that women entrepreneurs were severely affected by the global pandemic crisis, with two-third reporting a drop in revenue and less than 10% of women entrepreneurs reporting an increase in revenue (see Table 1). Moreover, almost a quarter of women entrepreneurs reported business model changes, with the transition or expansion to online services and sales stated as a clear opportunity, followed by over 15% identifying online marketing and better financial management and planning as needed going forwarded. Results from the second survey suggest that most women entrepreneurs were not well prepared for business interruption and for the impact of the pandemic on the marketplace (see Table 2).

**Table 1.** DIRI survey 1 results on COVID-19 impact on women entrepreneurs.

Responses	*N*	Count	%
Revenue has decreased	86	58	67.4
Actions taken:			
Deferring or reducing executive pay	86	34	39.5
Delaying payment for all or part of vendor bills and loan obligations	86	31	36.0
Reducing employee hours	86	22	25.6
Closing down my business permanently	86	20	23.3
Moving all or some employees to remote status	86	18	20.9
Putting workers on furlough	86	16	18.6
Laying off employees	86	14	16.3
Reducing employee pay	86	12	14.0
Closing down my business temporarily	86	4	4.7
Considering alternative types of incentive compensation	86	6	7.0
Other: moving, cutting G&A, new offerings, COVID-19 support, unemployment filings, outside income, rehire vendors at lower rate	86	17	19.8
Preferred type of assistance:			
Tax relief	86	37	43
Low interest federal loans	86	33	38.4
Federal wage subsidies	86	33	38.4
Deferred rent payments	86	26	30.2
Deferred loan payments	86	24	27.9
Federal reimbursements for employee paid leave	86	21	24.4
Vendor credit	86	11	12.8
Other: none, dk, rent/utility subsidy	86	13	15.1
Opportunities identified:			
Online services	86	20	23.3
Online marketing	86	13	15.1
New markets	86	8	9.3
Funding sources	86	6	7.0
Crisis planning	86	5	5.8
Education and training	86	4	4.7
Remote work	86	3	3.5
Contract terms	86	3	3.5

*n* = 86; 65% USA-based firms; 40.5% in Professional and Consumer Services; 74.1% with < US$1 million revenue; 93.9% with < 50 employees; 26.7% less than five years; 8.6% VC-funded and 34.4% ‘essential services’.

**Table 2.** DIRI survey 2 results on COVID-19 impact on women entrepreneurs.

Responses	*N*	Count	%
Business preparation:			
Cash-on-hand to cover three months or more of payroll and expenses	74	31	41.9
Good access to business support services and crisis guidance	74	20	27.0
Financial reports for YE2019 and YTD2020	74	19	25.7
Up-to-date on account receivables	74	18	24.3
Accounts with lender on SBA lending programme list	74	16	21.6
2020 sales forecast easily adjusted for estimating potential losses	74	16	21.6
Sufficient inventory to support operations for three months	74	13	17.6
Cost-cutting actions taken:			
Cutting office expenses	74	34	45.9
Reducing marketing spend	74	32	43.2
Freezing all hiring	74	24	32.4
Reducing salaries/hours	74	19	25.7
Renegotiating vendor contracts	74	18	24.3
Postponing new product launches	74	15	20.3
Postponing R&D investments	74	10	13.5
Deferring lease payments	74	8	10.8
Laying off employees	74	7	9.5
Terminating a lease early	74	4	5.4
Temporary business model adjustments:			
Offering virtual office visits	74	15	20.3
Streaming classes or services	74	15	20.3
Marketing/promoting in a different way	74	15	20.3
Offering new products/services	74	9	12.2
Other	74	14	(< 5.0)
Permanent business model adjustments:			
Offering new products/services	74	30	40.5
Marketing/promoting in a different way	74	25	33.8
Streaming classes or services	74	11	14.9
Offering virtual office visits	74	6	8.1
Other	74	12	(< 5.0)
Business relief programmes applied to:			
SBA Emergency Disaster Loan	74	25	33.8
Paycheck Protection Programme	74	30	40.5
Expected time to recovery:			
≤ one year	58	25	43.1
> one year	58	20	34.5
Don’t know	58	13	22.4

**SBA: Small business administration.**

*n* = 74; 92% USA-based firms; 58% in Finance, Professional Services and Wholesale/Retail Trade; 76.4% with < US$1 million revenue; 21% solopreneurs; 76% with < 50 employees; 49% less than five years; 8% VC-funded and 20% ‘essential services’.

The data from these surveys show that women entrepreneurs are clearly traumatised by the shock of the COVID-19 crisis. In fact, only about two-fifth of the women entrepreneurs in the second survey stated that they expected to resume normal operations with the year, while the rest said it would take more than one year, and/or that it was too soon to tell. While these women entrepreneurs have scrambled to adapt to the emergency conditions by making significant decreases in spending, they also recognised the opportunities offered by the COVID-19 pandemic and pivoted their business models in order to seize these opportunities. About half of all women entrepreneurs surveyed said they were offering new products/services and marketing in a different way, with more than a third reporting that these business model adjustments would be permanent. For example, about a third of the survey respondents reported offering virtual office visits and streaming classes or services, though the majority described these as temporary changes.

Contrary to assumptions predicting that women entrepreneurs will primarily adjust their business models to reduce risk, these findings suggest that women entrepreneurs moved very quickly to capture new business opportunities resulting from the crisis and change in circumstance. To quote one respondent:

Our online business, which has been a third of our revenue last year, has more than doubled – our wholesale business has evaporated. If we could refinance our existing debt I think we could ramp up our sales and come out of this transformed into an all-online business with higher margins.

## Women entrepreneurs and business model pivots: two COVID-19 case studies

While research on women entrepreneurs who pivot their business models to take advantage of the opportunities created from the COVID-19 pandemic is only just emerging, the popular press is replete with stories of talented women who are responding to the challenges of COVID-19 in creative and unique ways. Consider the case of Abigail Rose, founder of the recently launched media-technology company Blended Sense, which connects local businesses with creative individuals to produce digital assets. In March, when the city of Austin, Texas announced the cancellation of the popular festival South by Southwest (SXSW), Rose’s firm missed an opportunity to meet with potential new investors and customers. At the same time, Rose was just about to close on her first round of funding, a major US$100,000 deal. When the funding evaporated and between 40% and 45% of her customers suspended their subscriptions, Blended Sense moved quickly into survival mode, where the priority became combining productivity with cost-cutting. Rose said,

As the C-level team, the biggest challenge at the moment is to make sure on a daily basis that our team is motivated to go forward without the certainty of how long this drastic pivot is going to last for.

Rose also noticed that the COVID-19 pandemic created new opportunities for her firm. Local businesses were struggling with ways to engage their customers online, and Rose quickly tailored the company’s product to serve this new vertical. However, instead of offering a subscription-based business model, Rose switched to an *à la carte* model to make the price point more accessible to her customers. She also started hosting webinars for small businesses on how to use digital assets during the pandemic (Leprince-Rinquet, 2020). Rose’s two business pivots, first cost-cutting and then moving into a new line of business, coupled with her change in the pricing model, simultaneously reduced her risk of dissolution and created new discovery-based business opportunities (McGrath and Macmillan, 2009).

Consider also the case of Skida, which makes colourful limited edition hats, headbands, and neck warmers (www.skida.com). Founded by Corinne Prevot in 2008, in response to a lack of colourful ski headgear and based in Burlington VT, the team of 12 women and three men quickly realised the opportunity presented by the pandemic, and pivoted their business model from winter skiing gear to protective face coverings. The response from their customers was overwhelmingly positive. The company sold out of their first and second production runs of masks within 15 minutes of their going live on the Skida website.

However, unwilling to rest on their early success, Skida continued to innovate. Adopting a discovery-driven business model, Skida worked with local healthcare workers, designing a headband with buttons to be worn in conjunction with masks to help relieve the chaffing behind the ears that was caused by masks with elastic ear loops. Skida then went on to redesign their masks with ties made from colourful shoelaces, in lieu of the problematic elastic ear loops. Team members tested their new prototype design on trips to the grocery store and walks around Burlington, VT (www.skida.com). After receiving positive feedback, Skida worked on scaling production of the new masks. They also switched from only selling masks on their website to taking orders, which helped to avoid a scramble for the limited products, hence, improving both production planning and customer satisfaction.

The dynamic nature of discovery-driven business models implies that many of the constraints that are competitively important are unknown at the same time that critical resource allocation decisions are made (McGrath, 2010). Witness the pivots that Skida made, which are as follows: first, to masks that were constructed from repurposed ski headgear material, and second, to a better mask design. When firms adopt a discovery-driven approach, they change their focus from a pre-occupation with one application of resources, to a completely different use where those resources can exploit emerging opportunities. In addition to illustrating discovery-driven approaches, these cases challenge the dominant narrative on women entrepreneurs in the academic literature. Consider Orser and Riding (2018), who found that women are less likely to adopt information technology, or Mack et al. (2017), who found that social media could act as a barrier for women entrepreneurs. The case of Blended Sense pivoting during the COVID-19 crisis suggests that a women-led IT media business can creatively pivot its business model and adopt webinars to better serve its customers.

## Future research directions and conclusions

As exemplified by the case descriptions and the data from the DIRI, we find that the business model pivots made by women entrepreneurs were not simply offensive or defensive, thus, challenging the received crisis response stereotypical dichotomy in entrepreneurship research. In fact, in line with a discovery-driven approach, the business model pivots undertaken by women entrepreneurs simultaneously reduced risk and seized opportunities (McGrath, 2010). When entrepreneurs adopt a discovery-driven approach, they change their tactics from a pre-occupation on their previous and now defunct business model to a new model. They do this by focusing on their current resources and capabilities and identifying ways to apply these resources and capabilities to new opportunities.

Reflecting our focus on discovery-driven business model change, we propose a series of research questions to further our understanding of women-led business model pivots in a time of extreme exogenous shocks:

- How do women entrepreneurs lead their companies during a business model pivot?
- How do industry sector variations matter in business model pivots?
- For women entrepreneurs, what are the strategies used to balance the competing work–family demands created by the COVID-19 crisis?
- Are the pivoting strategies used by women entrepreneurs different in firms that have a business-to-business focus (B-to-B) as compared to those that have a business to consumer focus (B-to-C)?
- What are the outcomes of business model pivots? Have COVID-19 changes resulted in permanent changes in overall business goals? Do firms have an increased balance of social and economic outcomes?
- What role does digital technology play in business model pivots for women entrepreneurs? Are women entrepreneurs using technology to adjust to their business models?
- What impact has COVID-19 had upon the financial inclusion of women entrepreneurs? What types of business relief has been most useful for women entrepreneurs?
- What should policymakers and impact investors be focusing on when it comes to gendered policy responses to the COVID-19 pandemic?

In conclusion, in this commentary we argue that business model pivots cannot be fully understood without taking into consideration the economic and social structure in which businesses operate. In addition, we suggest that successful business model pivots, particularly in response to double economic shocks, such as those inflicted by COVID-19, must simultaneously reduce risk and seize opportunities (McGrath and Macmillan, 2009). However, we add a new dimension to this conversation by recognising that when it comes to economic considerations in times of crisis, gender issues are rarely considered. We hope that our comments provoke others to engage in research that not only explores pivots in business models, but also do so through the underrepresented gendered lens.
